# Clinical features of childhood granulomatosis with polyangiitis (wegener’s granulomatosis)

**DOI:** 10.1186/1546-0096-12-18

**Published:** 2014-05-26

**Authors:** Marek Bohm, Maria Isabel Gonzalez Fernandez, Seza Ozen, Angela Pistorio, Pavla Dolezalova, Paul Brogan, Giancarlo Barbano, Claudia Sengler, Marisa Klein-Gitelman, Pierre Quartier, Anders Fasth, Troels Herlin, Maria Teresa R A Terreri, Susan Nielsen, Marion A J van Rossum, Tadej Avcin, Esteban Rodolfo Castell, Ivan Foeldvari, Dirk Foell, Anuela Kondi, Isabelle Koné-Paut, Rolf-Michael Kuester, Hartmut Michels, Nico Wulffraat, Halima Ben Amer, Clara Malattia, Alberto Martini, Nicolino Ruperto

**Affiliations:** 1 Istituto Giannina Gaslini Pediatria II - Reumatologia, PRINTO, Genova, Italy; 2 Charles University in Prague, 1st Medical Faculty and General University Hospital in Prague, Prague, Czech Republic; 3 Department of Pediatric Rheumatology and Nephrology, Hacettepe University Children’s Hospital, Ankara, Turkey; 4 Istituto Giannina Gaslini, Servizio di Epidemiologia e Biostatistica, Genova, Italy; 5 Department of Rheumatology, Great Ormond St Hospital, NHS Foundation Trust, London, UK; 6 Istituto Giannina Gaslini, Nefrologia, Genova, Italy; 7 Department of Pediatrics, Division of Pneumology and Immunology, Charité Universitätsmedizin Berlin, Berlin, Germany; 8 Ann & Robert H. Lurie Children’s Hospital of Chicago, Chicago, IL, USA; 9 Université Paris-Descartes, Institut IMAGINE, Hôpital Necker-Enfants Malades, Centre de référence national pour les Arthrites Juveniles, Unité d’Immunologie, Hématologie et Rhumatologie Pediatrique, Paris, France; 10 Department of Pediatrics, The Queen Silvia Children’s Hospital, University of Gothenburg, Göteborg, Sweden; 11 Department of Pediatrics, Skejby Sygehus, Aarhus University Hospital, Pediatric Rheumatology Clinic, Aarhus, Denmark; 12 Universidade Federal de São Paulo, Pediatrics, São Paulo, Brazil; 13 Juliane Marie Centret, Rigshospitalet, Pediatrisk klinik II, Copenhagen, Denmark; 14 Department of Pediatrics, Emma Children Hospital AMC, Amsterdam, Netherlands; 15 Department of Allergology, Rheumatology and Clinical Immunology, University Children’s Hospital, University Medical Centre Ljubljana, Ljubljana, Slovenia; 16 Hospital Dr Felipe Glasman, Rheumatolgy Section, Bahía Blanca, Buenos Aires, Argentina; 17 Hamburger Zentrum für Kinder- und Jugendrheumatologie, Klinikum Eilbek Hs.6, Hamburg, Germany; 18 Department of Pediatric Rheumatology and Immunology, University Children’s Hospital, Muenster, Germany; 19 Pediatric Department, University Hospital Centre, Tirana, Albania; 20 CHU Le Kremlin Bicêtre, APHP, University of Paris Sud, CEREMAI (Centre de référence national des maladies auto-inflammatoires, rhumatologie pédiatrique), Le Kremlin Bicêtre, Paris, France; 21 Asklepios Klinik Altona, Hamburg, Germany; 22 Kinderklinik Garmisch-Partenkirchen gGmbH, Deutsches Zentrum für Kinder- und Jugendrheumatologie, Garmisch-Partenkirchen, Germany; 23 Department of Pediatric Immunology and Rheumatology, Wilhelmina Kinderziekenhuis, Utrecht, Netherlands; 24 Benghazi Children Hospital – Benghazi, MUB - Rheumatology Clinic, Benghazi, Lybia; 25 Dipartimento di Pediatria, Università degli Studi di Genova, Genova, Italy

**Keywords:** Wegener’s granulomatosis, Granulomatosis with polyangiitis, Clinical study, Clinical picture of disease, Comparison with literature

## Abstract

**Background:**

Granulomatosis with polyangiitis (GPA), formerly known as Wegener’s granulomatosis (WG), belongs to the group of ANCA-associated necrotizing vasculitides. This study describes the clinical picture of the disease in a large cohort of GPA paediatric patients.

Children with age at diagnosis ≤ 18 years, fulfilling the EULAR/PRINTO/PRES GPA/WG classification criteria were extracted from the PRINTO vasculitis database. The clinical signs/symptoms and laboratory features were analysed before or at the time of diagnosis and at least 3 months thereafter and compared with other paediatric and adult case series (>50 patients) derived from the literature.

**Findings:**

The 56 children with GPA/WG were predominantly females (68%) and Caucasians (82%) with a median age at disease onset of 11.7 years, and a median delay in diagnosis of 4.2 months. The most frequent organ systems involved before/at the time of diagnosis were ears, nose, throat (91%), constitutional (malaise, fever, weight loss) (89%), respiratory (79%), mucosa and skin (64%), musculoskeletal (59%), and eye (35%), 67% were ANCA-PR3 positive, while haematuria/proteinuria was present in > 50% of the children. In adult series, the frequency of female involvement ranged from 29% to 50% with lower frequencies of constitutional (fever, weight loss), ears, nose, throat (oral/nasal ulceration, otitis/aural discharge), respiratory (tracheal/endobronchial stenosis/obstruction), laboratory involvement and higher frequency of conductive hearing loss than in this paediatric series.

**Conclusions:**

Paediatric patients compared to adults with GPA/WG have similar pattern of clinical manifestations but different frequencies of organ involvement.

## Background

Granulomatosis with polyangiitis (GPA), previously known as Wegener’s granulomatosis (WG), [1] is a necrotizing vasculitis affecting predominantly small vessels. This disease is typically associated with granulomatous inflammation, pauci-immune necrotizing glomerulonephritis, involvement of upper and lower respiratory tract, and with presence of anti-neutrophil cytoplasmic antibodies (ANCA).

The estimated annual incidence of the disease in adults is 1:100,000, and 90% of the patients are Caucasians [2]. In children, the estimated incidence is approximately 0.1:100,000 [3]. If untreated, mortality, within one year from diagnosis is 90%. Treatment usually consists of combination of corticosteroids and cyclophosphamide, and more recently rituximab to induce remission, followed by a maintenance phase with lower doses of corticosteroids combined with azathioprine or other disease modifying agents for several years. Despite treatment relapses are common and therapy related complications of significant concern [4-9].

The clinical and laboratory picture of GPA/WG was described in several large cohorts of predominantly adult patients [10-12] but there is paucity of paediatric data due to the rare occurrence of the disease in childhood [6,13,14]. Recently, new criteria for childhood GPA/WG have been established and validated by the European League Against Rheumatism/Paediatric Rheumatology International Trials Organisation/Paediatric Rheumatology European Society (EULAR/PRINTO/PRES) [15-17].

The aim of this project was to describe the clinical and laboratory features at presentation of childhood GPA/WG in a large international cohort of paediatric patients collected by PRINTO, and compare this series with other paediatric series and with adult patients with WG/GPA derived from the literature.

### Patients and methods

The PRINTO database contains data on 1398 patients with childhood vasculitides, with age at diagnosis ≤ 18 years, vasculitis diagnosis after year 2000, as previously described [16,17]. The database includes demographic data, clinical diagnosis ascertained by the treating physician and a comprehensive list of 70 signs/symptoms (predominantly categorical variables) in 12 broad organ-system categories, laboratory parameters, physician global assessment of disease activity on a 10 cm visual analogue scale (VAS), biopsy findings and imaging reports. Data have been collected both retrospectively and prospectively, before or at the time of diagnosis and at least 3 months later via standardized web-based case report forms.

For the purposes of this analysis, we extracted all patients fulfilling the c-GPA/WG EULAR/PRINTO/PRES classification criteria [15-17]. Patients with co-morbidities were excluded from the study. In brief, a patient is classified as childhood GPA/WG if at least three of the six following criteria are present: 1) histopathology (granulomatous inflammation); 2) upper airway involvement (nasal discharge or epistaxis/crusts/granulomata, nasal septum perforation or saddle nose deformity, sinus inflammation); 3) laryngo-tracheo-bronchial involvement; 4) pulmonary involvement by chest X-ray or CT; 5) ANCA positivity; 6) renal involvement.

The study was approved by the ethics committee of the Gaslini Hospital (Genoa, Italy) and by the ethics committees of all remaining participating centres and informed consent was obtained from parent(s) as required by the national law in each participating country.

### Comparison with literature data (children and adults)

A PubMed (http://www.ncbi.nlm.nih.gov/pubmed/) search was used to retrieve studies, which included patients with GPA/WG (both children and adults), and included both prospective and retrospective cases describing the clinical picture of at least 50 patients. Search terms were “(((Granulomatosis AND (Wegener OR Wegener’s OR polyangiitis)) OR (necrotizing AND vasculitis))) AND (group OR cohort OR patients) AND (prospective OR prospectively OR retrospective OR retrospectively) AND demographic AND age AND clinical AND study” in English.

### Statistical analysis

The quantitative demographic, clinical and laboratory data were reported as median (1^st^ and 3^rd^ quartile); the categorical data were reported as absolute frequencies and as percentage frequencies of measured subjects.

The data were entered in an Access XP database and analysed with Statistica 6.0 (StatSoft, Inc).

## Findings

A total of 56/67 patients fulfilling the EULAR/PRINTO/PRES childhood GPA/WG classification criteria were identified; the remaining 11 patients were excluded for the following reasons: 1 age at onset > 18 years, 1 for the presence of other co-morbid condition, 7 excluded by consensus evaluation, 4 not fulfilling the EULAR/PRINTO/PRES as previously described [16,17]. Eighty-two% were Caucasians and 68% females, with median age at the time of disease onset of 11.7 years (1^st^ and 3^rd^ quartile 8.5-14.5 years) and with median delay in diagnosis of 4.2 (1.0-9.6) months.

The Table 1 shows in the first column the frequencies of symptoms before/at the time of diagnosis. The most frequent organ systems involved before/at the time of diagnosis were ears/nose/throat (ENT) (91%), followed by constitutional (malaise, fever, weight loss) (89%), respiratory (79%), mucosa and skin (64%), musculoskeletal (59%), and eye (35%). As indicated in the second column, most of the signs/symptoms present at the time of diagnosis improved when evaluated at least 3 months after diagnosis with the exception of some related to ENT **(**nasal septum perforation/saddle nose/auricular chondritis, subglottic stenosis/hoarseness/stridor), pulmonary (wheeze or expiratory dyspnoea, pulmonary nodules or cavities), and eye (conjunctivitis, keratitis, blepharitis) involvement which remained substantially unchanged (data not shown).

**Table 1:** Frequency of signs/symptoms (number of patients/%) at the time of diagnosis: comparison with published series

	**Paediatric cohorts**		**Adult cohorts**		
Signs/symptoms*	**Current cohort 2010 (N = 56]**	**Cabral et al., 2009**[6]**(N = 65]**	**Koldingsnes, 2000**[10]**(N = 55]**	**Mahr, 2008**[11]**(N = 180]**	**Stone, 2010**[7]**(N = 197)**
**Female**	**38 (68%)**	**41 (63%)**	**21 (38%)**		**99 (50%)**
**Constitutional**	**50 (89%)**				**120 (61%)**
Malaise	45/55 (82%)	58 (89%)	47 (85%)		
Fever	35 (62%)	35 (54%)			
Weight loss	30/52 (58%)	28 (43%)		12 (7%)	
**Mucosa and skin**	**36 (64%)**	**23 (35%)**	**17 (31%)**		
**Eye**	**19 (34%)**	**24 (37%)**	**21 (38%)**		
Red eye: conjunctivitis, keratitis, blepharitis	14/55 (25%)	6 (9%)		36 (20%)	
**Ears, nose, throat**	**51 (91%)**	**52 (80%)**	**44 (80%)**		**115 (58%)**
Oral/nasal ulcerations	28 (50%)	6 (9%)		26 (14%)	
Nasal discharge or recurrent epistaxis/crusts/granulomata	41 (73%)	42 (65%)		122 (68%)	
Nasal septum perforation/saddle nose/auricular chondritis	9 (16%)				
Chronic or recurrent sinus nasal inflammation	34 (61%)	39 (60%)		98 (54%)	
Chronic or recurrent otitis media/aural discharge	12/55 (22%)	9 (14%)		7 (4%)	
Conductive hearing loss	6/54 (11%)	7 (11%)		52 (29%)	
Subglottic stenosis/hoarseness/stridor	11 (20%)	9 (14%)		22 (12%)	
**Respiratory**	**44 (79%)**	**52 (80%)**	**33 (60%)**		**104** (53%)
Tracheal/endobronchial stenoses, obstruction	9 (16.1%)			11 (6%)	13 (7%)
Haemoptysis, alveolar haemorrhage	14/55 (25%)	29 (45%)		35 (19%)	50 (25%)
Pleuritis	7 (13%)	15 (23%)		16 (9%)	17 (9%)
Pulmonary nodules or cavities	17 (30%)	26/62 (42%)		67 (37%)	45 (23%)
Fixed lung infiltrates	26/55 (47%)	14/62 (23%)			
**Musculoskeletal**	**33 (59%)**				
Muscle pain/tenderness	33 (59%)	37 (57%)	35 (64%)		
Arthritis	16/55 (29%)	13 (20%)			
Arthralgia	29 (52%)				
**Other**					
Central nervous system	8 (14%)	16 (25%)	7 (13%)		39 (20%)
Gastro-intestinal system	9 (16%)	27 (42%)	3 (5%)		
Renal	38 (68%)	49 (75%)	42 (76%)		130 (66%)
**Laboratory/biopsy**					
ELISA MPO-ANCA	13/50 (26%)	15 (23%)			65 (33%)
ELISA PR3-ANCA	34/51 (67%)	44 (68%)			131 (67%)
Haematuria or red blood cells casts (RBC/hpf)	35 (63%)			77 (43%)	
Glomerulonephritis by renal biopsy	23/28 (82%)	34 (94%)			

Childhood *ANCA*, Cytoplasmic Anti-Neutrophil Cytoplasmic Antibodies; *MPO-ANCA*, myeloperoxidase-specific Anti-Neutrophil Cytoplasmic Antibodies; *PR3-ANCA*, proteinase 3-specific Anti-Neutrophil Cytoplasmic Antibodies; *RBC/hpf*, red blood cells per high power field.

Blank cells refers to data not available in the related publication.

*Blank fields refers to not available data.

When evaluated for ANCA positivity, at the time of diagnosis, 67% children were ELISA PR3 and 26% ELISA MPO ANCA positive which decreased to 39% and 19.5% respectively at the subsequent measurement at least 3 months later; ANCA immunofluorescence staining was positive in 82.7% at diagnosis and in 47.6% 3 months thereafter. Haematuria/proteinuria was present in > 50% cases at the time of diagnosis (with necrotizing pauci-immune glomerulonephritis in 23/28 [82.1%] biopsies available) and in >30% at least 3 months later.

The Table 1 also compares the present series (N = 56) with the paediatric series by Cabral (N = 65) [6] and with 3 adult case series (N = 55-180) [7,10,11]. The organ involvement of the present paediatric series was comparable with the other paediatric series with some exceptions; in our series we observed an higher frequency of conjunctivitis, oral/nasal ulceration, fixed lung infiltrates, but lower frequency of haemoptysis/alveolar haemorrhage, and gastrointestinal involvement compared with the study by Cabral et al. [6].

When compared to adult series, adult patients showed lower frequencies of constitutional (fever, weight loss), ENT (oral/nasal ulceration, chronic or recurrent otitis media/aural discharge), respiratory (tracheal/endobronchial stenosis, obstruction, haemoptysis/alveolar haemorrhage), and renal (haematuria or red blood cells casts) involvement, and a higher frequency of conductive hearing loss.

## Discussion

In this study, we have analysed the clinical features at the time of diagnosis of a large sample of children with GPA/WG whose data were retrieved from the PRINTO database and were collected on the occasion of the project that led to the validation of the EULAR/PRINTO/PRES criteria for the classification of childhood vasculitis [15-17].

Granulomatosis with polyangiits is very rare in children and, although it appears to be the same disease observed in adults, it is not known if childhood presentations are characterized by differences in the frequency of some clinical features well described in adults. Indeed, in other connective tissue diseases, such as systemic lupus erythematosus or juvenile dermatomyositis, the frequency of several clinical manifestations differed from those observed in adults [18,19].

We have therefore investigated the clinical and laboratory features present at the time of diagnosis in our series of 56 children and compared them with those of the only other large series of paediatric patients (N = 65) reported in the literature as well as with 3 large (N > 50) reported series of adult patients with GPA/WG.

In agreement with previous studies in children, we have found a gender disproportion, as 68% of our patients were females, [6] while in adult populations females are less often affected ranging from 29 to 50% [7,8,10].

In the present paediatric series, the disease was characterised by high frequency of constitutional symptoms, upper and lower respiratory tract, mucosa, skin and musculoskeletal involvement. The frequencies of symptoms typically decreased in the first few months after diagnosis, presumably due to the effect of treatment although data on treatment modalities were not collected in the database. Our series confirms the previous observation by Cabral et al that patients with time to diagnosis longer than 12 months have a higher frequency of ear, nose and throat and cutaneous symptoms and less renal symptoms in comparison with earlier diagnosed patients [6]. This likely reflects the non-specific nature of ENT and cutaneous features in GPA, that occur commonly in children and hence may not prompt clinicians to initially consider vasculitis as the cause. Out of 26 of our patients who underwent kidney biopsy, 82% had glomerulonephritis, similar to the finding by Cabral et al. (94.4% of children with biopsies available) [6]. In adult patients, the data were similar with 73.5% of all patients found with histological evidence of glomerulonephritis [20].

Comparing the paediatric and adult cohorts, while organ involvement, signs and symptoms were similar, there were differences in their frequencies at disease presentation. Adults had a lower frequency of constitutional, ENT and respiratory involvement; while hearing loss was more frequent in the adult population.

The current work is limited by the lack of information on treatment and disease outcomes. In addition, comparison of clinical and laboratory findings with literature data might be hampered by the lack of standardised definition and by differences in the time of evaluation in the different series. Moreover our study was likely hampered by inconsistencies in laboratory techniques (in particular in relation to ANCA testing), and by the exclusion of cases in this series who did not fulfil the new paediatric classification criteria for c-GPA. Indeed, we recognise that patients may present with limited forms of the disease, without initially fulfilling all criteria for classification and clinicians must remain vigilant in relation to this presentation in children.

In conclusion, paediatric patients compared to adults with GPA/WG have a similar pattern of clinical manifestations but present different frequencies of organ involvement.

### Key messages

Children with GPA/WG are predominantly females/Caucasians with a median onset age of 11.7 years.

Adult showed lower frequencies of female, constitutional, ears/nose/throat, respiratory, laboratory involvement and higher frequency of hearing loss.

Paediatric patients compared to adults have similar clinical manifestations but different frequencies of organ involvement.

## Competing interests

The authors declare that they have no competing interest.

## Authors’ contribution

We confirm that all the named co-authors have participated in the: conception and design, or analysis and interpretation of data drafting the article or revising it critically for important intellectual content, final approval of the version to be published. All authors read and approved the final manuscript.
